# Comparison of two hybrid sentinel node tracers: indocyanine green (ICG)-^99m^Tc-nanocolloid vs. ICG-^99m^Tc-nanoscan from a nuclear medicine and surgical perspective

**DOI:** 10.1007/s00259-023-06157-9

**Published:** 2023-03-17

**Authors:** Manon T. A. Vreeburg, Samaneh Azargoshasb, Danny van Willigen, Tom Molenaar, Matthias N. van Oosterom, Tessa Buckle, Leon J. Slof, Martin Klop, Baris Karakullukcu, Maarten Donswijk, Henk G. van der Poel, Fijs W. B. van Leeuwen, Oscar R. Brouwer, Daphne D. D. Rietbergen

**Affiliations:** 1 Department of Urology, The Netherlands Cancer Institute-Antoni van Leeuwenhoek Hospital, Amsterdam, The Netherlands; 2 Interventional Molecular Imaging Laboratory, Department of Radiology, Leiden University Medical Hospital, Leiden, The Netherlands; 3 Radiochemistry Facility, Department of Radiology, Leiden University Medical Hospital, Leiden, The Netherlands; 4 Instrumentele zaken ontwikkeling, facilitair bedrijf, Leiden University Medical Centre, Leiden, The Netherlands; 5 Department of Head and Neck Surgery, The Netherlands Cancer Institute-Antoni van Leeuwenhoek Hospital, Amsterdam, The Netherlands; 6 Department of Nuclear Medicine, The Netherlands Cancer Institute-Antoni van Leeuwenhoek Hospital, Amsterdam, The Netherlands; 7 Department of Radiology, Section Nuclear Medicine, Leiden University Medical Hospital, Leiden, The Netherlands

**Keywords:** Penile cancer, Head & neck melanoma, Fluorescence, Image-guided surgery, Hybrid tracer

## Abstract

**Background:**

Lymph node (LN) metastasis is a relevant predictor for survival in patients with a.o. penile cancer (PeCa), malignant melanoma. The sentinel node (SN) procedure comprises targeted resection of the first tumour-draining SNs. Here, the hybrid tracer indocyanine green (ICG)-^99m^Tc-nanocolloid has been used for several years to combine optical and nuclear detection. Recently, the resource of the nanocolloid precursor stopped production and the precursor was replaced by a different but chemically comparable colloid, nanoscan. Our aim was to study the performance of ICG-^99m^Tc-nanoscan compared to ICG-^99m^Tc-nanocolloid from a nuclear and surgical perspective.

**Methods:**

Twenty-four patients with either PeCa or head-and-neck (H&N) melanoma and scheduled for a SN procedure were included. The initial group (*n* = 11) received ICG-^99m^Tc-nanocolloid until no longer available; the second group (*n* = 13) received ICG-^99m^Tc-nanoscan. Tracer uptake was assessed on lymphoscintigraphy and single-photon emission (SPECT). Intraoperatively, SNs were identified using gamma tracing and fluorescence imaging. Ex vivo (back-table) measurements were conducted to quantify the fluorescence emissions. Chemical analysis was performed to compare the ICG assembly on both precursors.

**Results:**

The mean tracer uptake in the SNs was similar for ICG-^99m^Tc-nanocolloid (2.2 ± 4.3%ID) and ICG-^99m^Tc-nanoscan (1.8 ± 2.6%ID; *p* = 0.68). 3 SNs (interquartile range (IQR) 3–4) were detected on lymphoscintigraphy in PeCa patients receiving ICG-^99m^Tc-nanoscan compared to 2 SNs (IQR 2–3) in PeCa patients receiving ICG-^99m^Tc-nanocolloid (*p* = 0.045), no differences were observed in H&N patients. Back-table measurements of resected SNs revealed a lower total fluorescence intensity in the ICG-^99m^Tc-nanoscan group (24*10^9^ arbitrary units (A.U) IQR 1.6*10^9^–14*10^9^ in the ICG-^99m^Tc-nanocolloid group versus 4.6*10^9^ A.U. IQR 2.4*10^9^–42*10^9^ in the ICG-^99m^Tc-nanoscan group, *p* = 0.0054). This was consistent with a larger degree of “stacked” ICG observed in the nanoscan formulation. No tracer-related adverse events were reported.

**Conclusions:**

Based on this retrospective analysis, we can conclude that ICG-^99m^Tc-nanoscan has similar capacity for SN identification as ICG-^99m^Tc-nanocolloid and can safely be implemented in SN procedures.

**Supplementary Information:**

The online version contains supplementary material available at 10.1007/s00259-023-06157-9.

## Introduction

The pathway of metastases in patients with many solid tumours (e.g. breast cancer [1], malignant melanoma [2] and penile cancer (PeCa) [3]) often primarily starts with lymphatic dissemination before distant metastases occur. In the case of lymph node (LN) involvement, the survival rate of patients decreases tremendously [4, 5]. In the case of malignant melanoma and PeCa, this makes LN metastases a relevant predictive factor for prognosis and overall survival [6, 7]. Generally, macro-metastases can be identified via palpation and/or preoperative imaging (e.g. ultrasound, CT, PET/CT or MRI). However, 13–16% of the clinically node negative (cN0) patients are reported to have subclinical LN metastases [8]. For the timely detection of LNs harbouring such subclinical metastases, additional treatment is necessary, which often included surgical nodal sampling techniques based on (templated) LN dissections. Yet, lymphadenectomy is associated with significant morbidity, e.g. edema and infection, induced by the disruption and removal of healthy and critical lymphatic anatomies.

To reduce morbidity, the sentinel node (SN) procedure has been introduced, aiming at targeted resection of the first tumour-draining SNs [9]. The procedure found its origin in staging of patients with malignant melanoma [10] and PeCa [11] and is still routinely implemented today in these indications [2, 12]. Preoperative nuclear imaging based on radiocolloids makes the heart of the SN identification process. Here, the lymphatic drainage can be mapped using lymphoscintigraphy and single-photon emission computed tomography and combined with low-dose computed tomography (SPECT/CT). Intraoperatively, the radioactive “hot” SNs can be acoustically traced using gamma-detection probes [13–15]. As surgery is a vision-oriented discipline, optical dyes such as blue dye and the near-infrared fluorescent dye indocyanine green (ICG) have been introduced in the operating room to complement the radioguidance procedure [12]. The hybrid SN tracer ICG-^99m^Tc-nanocolloid was introduced to merge the use of the radio- (^99m^Tc-nanocolloid) and optical-guidance (ICG) [16, 17]. This hybrid approach has been extensively validated during different SN indications: breast [18], melanoma [19, 20], penis [12], vulva [21], prostate [22], H&N [23, 24], cervix [25] and oesophagus [26]. Despite the good results and widespread implementation of ICG-^99m^Tc-nanocolloid (*N* > 2000 patients), a factory closing meant the resource for nanocolloid, a human serum albumin-based colloid, has recently become unavailable.

Various studies prospectively compared the performance of ICG-^99m^Tc-nanocolloid to other radiocolloids. Brouwer et al. studied twenty-five patients who received both ^99m^Tc-nanocolloid and ICG-^99m^Tc-nanocolloid in consecutive order, to indicate concordance in performance of these tracers [17]. A comparison between ICG-^99m^Tc-nanocolloid and ^99m^Tc-Senti-Scint, a larger particle-based radiocolloid revealed a size-related difference in SN identification between the two tracers [27]. Others have used retrospective setups to compare ^99m^Tc-nanocolloid to ^99m^Tc-tilmanocept [28], ^99m^Tc-nanoscan [29], ^99m^Tc-Albu-Res (also a larger particle-based radiocolloid) [30] and ^111^In-labelled leukocytes [31]. Thus, such retrospective approaches also provide a valid means of accessing concordance.

Now, ^99m^Tc-nanoscan has replaced ^99m^Tc-nanocolloid as the available radiocolloid for SN procedures in the European market. Both agents are considered as nanocolloids by the EMEA and have similar particle size (95% of both human albumin colloidal particles have a diameter of ≤ 80 nm). While ^99m^Tc-nanoscan is considered pharmaceutically equal to ^99m^Tc-nanocolloid, subtle formulation differences warrant evaluation of the performance of ICG-^99m^Tc-nanoscan compared to ICG-^99m^Tc-nanocolloid. We pursued such a comparison based on equally sized retrospective cohorts of patients undergoing SN procedures for PeCa and H&N melanoma. Using pre-, intra- and postoperative imaging findings, we assessed the concordance between the two hybrid tracers from a nuclear medicine and surgical perspective.

## Methods

### Patient selection

In this retrospective study, twenty-four patients who were scheduled for a SN procedure at a single high-volume European centre between January and July 2022 were included. All patients were clinically node negative (cN0) at the time of the SN procedure. Patients underwent SN procedures, following the hybrid tracer injection, as part of routine clinical care. The study was approved by the institutional review board of the NKI-AVL. The initial group of 11 patients (*n* = 5 PeCa, *n* = 6 H&N melanoma) received ICG-^99m^Tc-nanocolloid (GE Healthcare BV, Leiderdorp, The Netherlands). The second group of 13 patients received the hybrid tracer ICG-^99m^Tc-nanoscan (*n* = 8 PeCa, *n* = 5 H&N melanoma, GE Healthcare BV).

### Preoperative procedure and image analysis

All patients underwent a routine SN procedure as previously described [13]. In short, the hybrid tracer was injected intracutaneously around the tumour in 3–4 depots on the same day (*n* = 19, 76%, 1-day protocol) or the day before surgery (*n* = 5, 20%, 2-day protocol), both was standard procedure. The median dose was 108 MBq (IQR 106–140 MBq) and 213 MBq (IQR 164–227 MBq), respectively. Dynamic and static planar lymphoscintigraphy were performed directly (0–10 min), at 15 min and 2 h after the injection, followed by SPECT/CT (Symbia T; Siemens, Erlangen, Germany). The visualization of SNs and higher echelon nodes was assessed on lymphoscintigraphy and SPECT/CT. Inguinal lymphatic drainage of the penis was based on the zones of Daseler’s classification [3] and the drainage of the H&N melanoma was based on the level system of cervical LN classification [32]. Lymphoscintigraphy images were analysed by two independent readers with more than 10 years of experience. To calculate the percentage of tracer uptake in SNs (percentage injected dose (%ID), regions of interest (ROIs), using Horos an open-source DICOM-viewer software (Lesser General Public License, Annapolis, USA), were drawn at the injection site and the SNs using late planar lymphoscintigraphy images, as described earlier [28].

### Intraoperative procedure

Prior to incision, the surgeon used the overview images of the SNs provided by a portable gamma-camera (Sentinella; OncoVision, Valencia, Spain) to guide the skin incisions. Next, SNs were pursued using the acoustic guidance provided by a gamma probe (Neoprobe; Johnson & Johnson Medical, Hamburg, Germany) followed by SN visualization using a fluorescence camera (FIS-00; Hamamatsu Photonics, Hamamatsu, Japan). Following SN excision, the portable gamma and fluorescence camera were used to check for residual activity. If activity was found, it was considered an additional SN and was also excised. Adverse events were reported, if present.

### Ex vivo analysis

Ex vivo, SNs were separated from surrounding fatty tissue to conduct further evaluation by fluorescence imaging with the FIS-00 camera. The specimens were placed in a custom black box that disabled ambient light infiltration. Identical camera settings, focus and at a fixed distance (15 cm from lens to specimen) were used for all specimens. The fluorescence intensity in the obtained images was determined using a colour-based image segmentation algorithm created in MATLAB®, as described by Azorgoshash et al. [33].

### Histopathological examination

After surgery, all excised SNs were fixed in formalin, bisected, embedded in paraffin and cut at a minimum of 6 levels at 50- to 150-μm intervals. Pathologic evaluation included haematoxylin-eosin and immunohistochemically staining.

### Quantification of bound ICG

To determine the non-covalent ICG binding, nanocoll and nanoscan precursors were both dissolved in 1.95 mL saline (sterile) followed by short centrifugation to recover the complete amount of protein. Hereafter 0.50 μL of ICG in water for injection (5 mg/mL) was added and the mixture was agitated shortly and was then left to stand for 1–2 min. Subsequently, 1 mL of the solution was brought onto a MidiTrap G-25 size exclusion chromatography column (equilibrated with > 15 mL saline). Elution was performed using 1.5 mL saline per fraction. Lastly, unbound ICG was eluted of the column using 15 mL of MiliQ. The collected fractions were sampled and analysed using absorbance and fluorescence spectroscopy. The absorption in the fractions was corrected for dilution (Fig. 4A).

### Statistics

Statistical significance for differences between the two radiocolloids was established via an independent-sample Mann-Whitney *U* test with SPSS statistical software for Windows (IBM SPSS Statistics, version 27).

## Results

### Preoperative imaging findings

Hybrid tracer injection occurred both in a one- (*n* = 19 (PeCa *n* = 10, H&N *n* = 9)) or two-day protocol (*n* = 5 (PeCa *n* = 3, H&N *n* = 2)). The use of the protocol was distributed among the tracers as follows: ICG-^99m^Tc-nanoscan group (1 day *n* = 12, 2 days *n* = 1) and ICG-^99m^Tc-nanocolloid group (1 day *n* = 7, 2 days *n* = 4).

No tracer-related adverse events were reported for either tracer. The majority of inguinal lymph drainage (85%) in PeCa patients was identified superior to the saphenous-femoral junction (SFJ), whereas in H&N melanoma most of the lymphatic drainage was seen in the upper jugular region (level II) and the parotid region (64%, supplementary Table 1). In PeCa patients, a significantly higher median number of SNs was identified on lymphoscintigraphy in the ICG-^99m^Tc-nanoscan group compared to the ICG-^99m^Tc-nanocolloid group, 3 SNs (IQR 3–4) versus 2 SNs (IQR 2–3), respectively, *p*-value = 0.045. No difference could be observed in H&N patients (ICG-^99m^Tc-nanoscan: 1 SN, IQR 0.5–2.5 versus ICG-^99m^Tc-nanocolloid: 2 SNs, IQR 2–2.5), *p*-value = 0.33. The median higher echelon nodes per hybrid tracer did not differ between the 2 indications (penile: *p*-value 0.83; H&N: *p*-value: 1.0 (see Table 1). In one H&N patient in the ICG-^99m^Tc-nanocolloid group, two SNs were only detected by lymphoscintigraphy as the overwhelming signal from the nearby injection site blurred the SPECT/CT image. Non-visualization occurred in one patient with head-and-neck melanoma, in the ICG-^99m^Tc-nanoscan group (no SN surgery was performed in this patient).

**Table 1:** Pre-, intra- and postoperative findings of SN procedures in patients with PeCa and H&N melanoma

	ICG-99mTc-nanocolloid *n* = 11	ICG-99mTc-nanoscan *n* = 13
*Preoperative findings*		
Age at surgery (yr), median (IQR)	71 (60–75)	73 (63–75)
Primary tumour		
H&N melanoma, *n*	6	5
PeCa, *n*	5	8
Lymphoscintigraphy		
SN		
H&N melanoma, *n* (median, IQR)	13 (2, 2 – 2.5)	7 (1, 0.5 – 2.5)
PeCa, *n* (median, IQR)	12 (2, 2 – 3)	27 (3, 3 – 4)
HE		
H&N melanoma, *n* (median, IQR)	11 (2, 0 – 4)	8 (2, 0 – 3.5)
PeCa, *n* (median, IQR)	15 (3, 2 – 4)	28 (3, 2 – 4)
SN on SPECT/CT (% of SN on lymphoscintigraphy)	92% (23/25)	100% (34/34)
Non-visualization on preoperative imaging, *n*	0	1
*Intraoperative findings*		
Interval between injection and OR in h, median (IQR)	5.5 (4.5 – 19.0)	5.0 (4.4 – 6.0)
Basins per patient, mean	2.2	1.7
Intraoperative SNs visualized by gamma probe	100% (38/38)	100% (47/47)
Intraoperative SNs visualized by fluorescence camera	97% (37/38)	98% (39/40)
Additional SNs found with portable gamma-camera, *n*	4	6
Excised SNs, *n* (median, IQR)	38 (3, 2 – 4)	47 (3, 2 – 5.5)
*Postoperative findings*		
Tumour-positive nodes, *n*	6	3
Tumour-positive nodes detected by gamma probe, %	100%	100%
Tumour-positive nodes visualized by fluorescence camera in vivo, %	83% (5/6)	100% (2/2)
Tumour-positive nodes in additionally found SN with portable gamma-camera, *n*	0	0

*H&N* head-and-neck, *HE* high echelon node, *ICG* indocyanine green, *OR* operation room, *SN* sentinel node

**Fig. 1:**
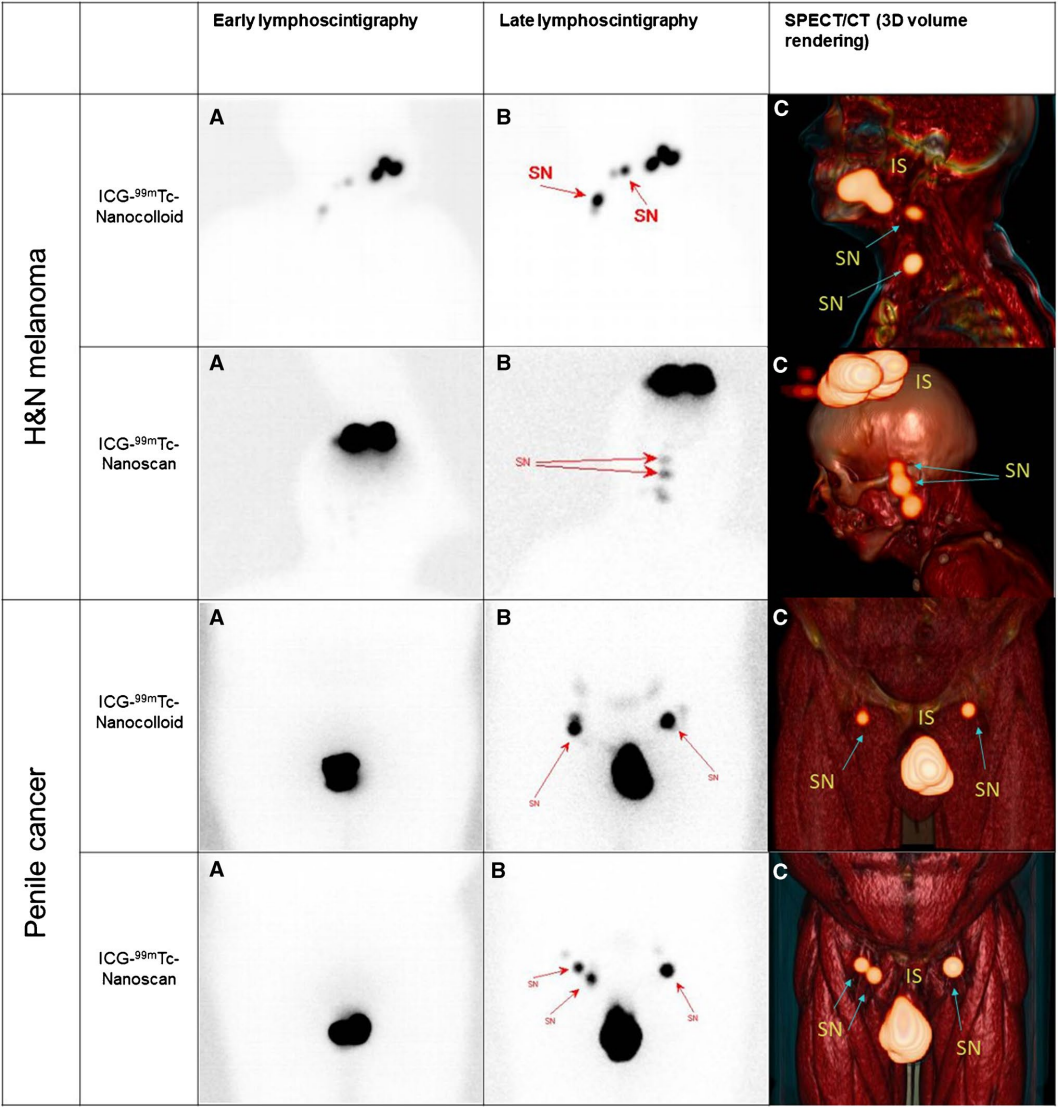
Examples of preoperative imaging in patients with head-and-neck melanoma (first two rows where first row is ICG-^99m^Tc-nanocolloid) and PeCa (last two rows, where first row is again ICG-^99m^Tc-nanocolloid). From *left* to *right* **A** early planar lymphoscintigraphy; **B** lymphoscintigraphy after 2 h with the location of the SNs (*arrows*); **C** a 3D volume rendering of the SPECT/CT (*arrows*). SN sentinel node, IS injection site, H&N head-and-neck

**Fig. 2:**
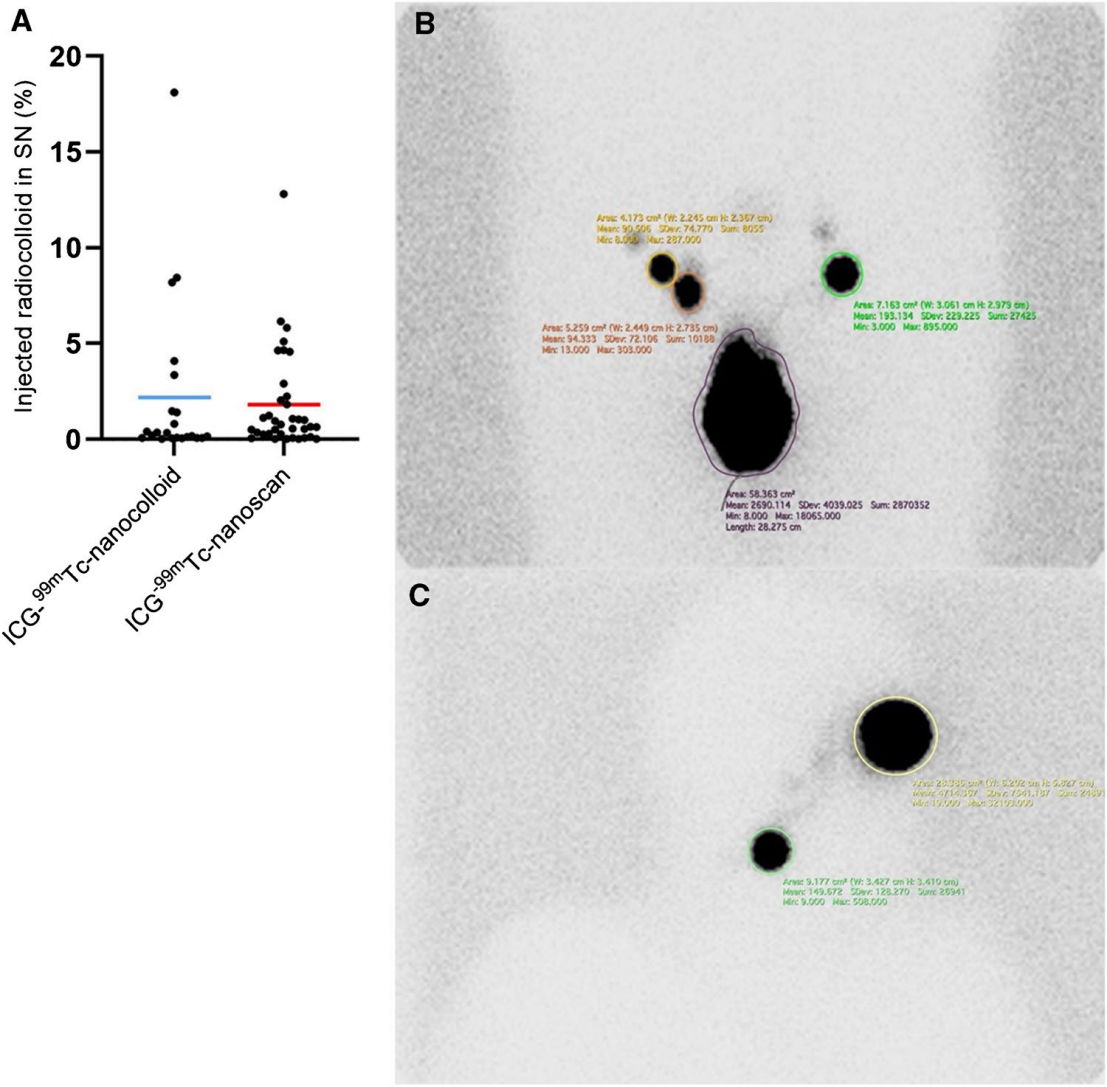
The uptake of injected radiocolloid in SNs on planar lymphoscintigraphy. **A** There is no difference in uptake when ICG-^99m^Tc-nanocolloid (*blue line*) and ICG-^99m^Tc-nanoscan (*red line*) are compared. **B** Example of analyses in Horos in a patient with PeCa; ROIs around the injection site (*purple circle*) and SNs (*green, orange and yellow circles*). **C** And the analysis of a patient with melanoma; ROIs around the injection site (*yellow circle*) and SNs (*green circle*)

As emphasized in Figs. 1 and 2, the tracer performance at preoperative imaging was similar. The percentage of radiocolloid uptake in the SNs on preoperative lymphoscintigraphy proved equal in the two groups with a mean of 2.2% ID (with a standard deviation (±) of 4.3% ID) in the ICG-^99m^Tc-nanocolloid group and 1.8% ID (± 2.6%ID) in the ICG-^99m^Tc-nanoscan group (*p*-value = 0.68).

### Intraoperative findings

None of the surgeons reported a difference in the intraoperative SN visualization, both with fluorescence and gamma tracing, between the two tracers. During surgery, the portable gamma-camera and gamma probe allowed detection of all SNs in both groups. Fluorescence imaging was used for localization of the SNs in the surgical field. Eight of the total 85 excised SNs were superficial nodes making them easy to identify without using the FIS-00 camera (ICG-^99m^Tc-nanocolloid: *n* = 1; ICG-^99m^Tc-nanoscan: *n* = 7, 10% overall). In the ICG-^99m^Tc-nanocolloid group, 34% of the surgically removed radioactive and fluorescent LNs were not detected as SN hot spot on planar lymphoscintigraphy, most likely these were higher echelon nodes (Table 1); this is in line with previous literature [34]. Whereas in the ICG-^99m^Tc-nanoscan group, this was 28% (*p*-value = 0.24).

**Fig. 3:**
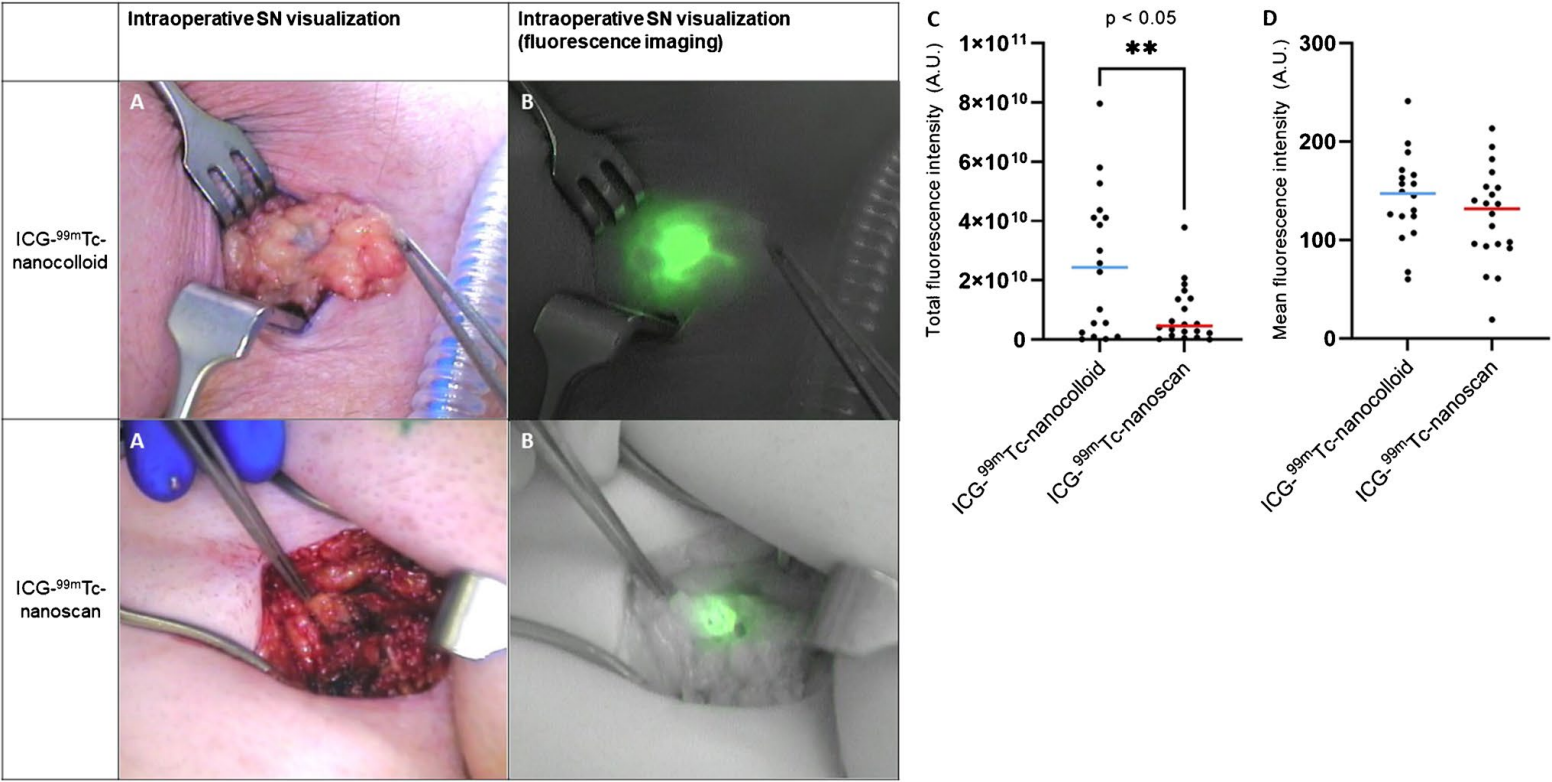
Intraoperative findings. *Left:* examples of intraoperative SN visualization with white light (**A**) and with a near-infrared fluorescence camera (**B**) in patients with PeCa. *Right:* the fluorescence intensity measured on the ex vivo images of excised SNs. Illustrated are the total (**C**) and the mean (**D**) fluorescence intensity measurements of ICG-^99m^Tc-nanocolloid (*blue line*) and ICG-^99m^Tc-nanoscan (*red line*). A.U. arbitrary units

Resected samples were subjected to back-table fluorescence imaging, after removal of surrounding fatty tissue. Back-table measurements indicated all radioactive nodes were fluorescent and vice versa. Figure 3 C shows a significant difference in relative total fluorescence intensity with a median of 4.6*10^9^ arbitrary units (A.U.) in the ICG-^99m^Tc-nanoscan group (IQR 2.4*10^9^ A.U.–42*10^9^ A.U.) and 24*10^9^ A.U. in the ICG-^99m^Tc-nanocolloid group (IQR 1.6*10^9^ A.U.–14*10^9^ A.U.), *p*-value = 0.0054. Comparisons based on mean intensity did not show significance (Fig. 3D, *p*-value = 0.22). The indication for surgery, PeCa or H&N melanoma, did not influence these results. When comparing the highest radioactive counts of the SNs with the intraoperative gamma probe, a higher radioactivity was found for the 1-day protocol (mean (SD) 4425 (6587) gamma counts) than in the 2-day protocol (618 (594) gamma counts), *p*-value = 0.02. No difference was found between the two hybrid tracers (*p*-value = 0.97).

### Pathology

In the ICG-^99m^Tc-nanocolloid group, 17% of the excised nodes appeared tumour positive (*n* = 4 H&N melanoma; *n* = 2 PeCa), whereas 8% in the ICG-^99m^Tc-nanoscan group (*n* = 2 H&N melanoma; *n* = 1 PeCa), *p*-value = 0.65. No differences in radioactive signal were found when comparing tumour-positive SNs (N+, median 1500 counts, IQR 291–4400 counts) and tumour-negative SNs (N-, median 1400 counts, IQR 322–3750 counts), *p*-value = 0.58. With a median follow-up of 5 months (IQR 4–6), there were no false-negative procedures so far with either tracer.

### Difference in ICG loading between nanocolloid and nanoscan

**Fig. 4:**
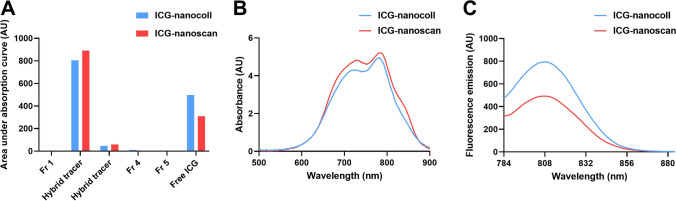
Difference in ICG loading in a direct chemical comparison between ICG nanocoll and ICG nanoscan. **A** ICG concentration in nanocoll (*blue*) and nanoscan fractions (*red*) based on the area under curve of the absorption curves per fraction, suggesting slightly more ICG binding with ICG nanoscan. **B** Absorption spectrum measurement of fraction 2 (containing the bulk of the hybrid tracer) of ICG nanocoll (*blue line*) and ICG nanoscan (*red line*), suggesting more ICG “stacking” with ICG nanoscan (stacking peak visible around 782 nm). **C** Fluorescence emission of fraction 2 of ICG nanocoll (*blue line*) and ICG nanoscan (*red line*), showing lower emission of ICG nanoscan. Fr fraction number

In a direct chemical comparison between ICG nanocoll and ICG nanoscan, ICG showed slightly more non-covalent binding to nanoscan as compared to nanocolloid (fraction 2 Fig. 4A). The absorption spectrum measurements on the hybrid tracer fraction indicated a larger degree of “stacked” ICG in the case of nanoscan (absorption peak at 782 nm) (Fig. 4B). In line with this finding, the fluorescence emission intensity of ICG nanoscan was lower compared to ICG nanocoll (Fig. 4C). It is unclear how these chemical analyses relate to the clinical situation.

## Discussion

The current retrospective comparison of ICG-^99m^Tc-nanocolloid and ICG-^99m^Tc-nanoscan indicates that the performance in patients of the two hybrid tracers is highly similar, even though chemical analysis indicated some differences (Fig. 4A). Hence, the newly implemented ICG-^99m^Tc-nanoscan seems fit to replace ICG-^99m^Tc-nanocolloid in daily clinical practice.

In agreement with literature, we observed a consistent performance based on preoperative imaging [2, 12] (Figs. 1 and 2). With regard to the intraoperative use of fluorescence imaging, in both groups, the fluorescent signature equally helped to pinpoint the nodal locations in the surgical field (Fig. 3). As fluorescence light is strongly attenuated by tissue [35], the experienced surgeons involved in the study are used to encountering a range of fluorescent signal intensities during the procedure and hence tend to rely on the ability to distinguish a specific signal intensity in the surgical field rather than the intensity of the fluorescent source [33]. As such, the small but significant difference observed during ex vivo analysis did not seem to be related to the procedural accuracy.

Spectral analysis indicates that while both precursors are considered to be part of the ^99m^Tc-nanocolloid family, the formulations used for nanocolloid and nanoscan impact the complexation of ICG. Specifically, absorbance spectra of the nanoscan-derived product indicate a higher ICG loading rate but also indicate a near 50% dye stacking [36]. As dye stacking leads to fluorescence quenching, this could negatively affect the in vivo fluorescence brightness. Nevertheless, it remains difficult to directly relate findings in the controlled environment of a chemistry lab with the complex biology encountered in patients; patients tend to vary substantially in tissue physiology and/or composition. In vivo, there is an excess of human serum albumin, which is a competitive binder for ICG [37]. Hence, a higher ICG loading rate could theoretically result in a rise in discordance between fluorescence and nuclear nodal detection. That said, we observed full concordance between the two, meaning all radioactive SNs were fluorescent and reversed.

In the ICG-^99m^Tc-nanoscan group, a higher percentage of patients has received the tracer injection and the SN procedure on the same day (1-day protocol, 92%), compared to the ICG-^99m^Tc-nanocolloid group (64%). We have found the SN radioactivity to be higher for the 1-day protocol when compared to the 2-day protocol, which is in line with the results of Dimopoulos et al. [38]. This could indicate that higher gamma counts are likely to cause nearby nodes to have detectable radioactivity, which may explain the lower rate of excised nodes that were not detected on imaging, 28% for the ICG-^99m^Tc-nanoscan group compared to 34% for the ICG-^99m^Tc-nanocolloid group, although this statement should be interpreted with caution given the small numbers.

The absence of adverse events is a key evaluation parameter for testing new chemical entities in humans. Both tracers are build up out of registered products, thereby reducing the chance that adverse events occur. Previous mid- to long-term follow-up studies with ICG-^99m^Tc-nanocolloid did not indicate adverse events [12, 23]. While relatively short-term and in a small patient cohort, the current analysis suggests that ICG-^99m^Tc-nanoscan also does not induce adverse events. Carefully indicating the “new” hybrid tracer is also safe to use in humans.

The limitations of the present analysis are, in addition to its retrospective nature, the fact that we only included a limited number of patients. The restriction herein is the ex vivo analysis that we performed to quantify the fluorescence intensity. Overall, the observed drainage patterns and pre- and intraoperative SN identification rates (see Table 1) seem to be in line with what has been extensively reported for ICG-^99m^Tc-nanocolloid. Dell’Oglio et al. assessed the performance of ICG-^99m^Tc-nanocolloid for PeCa SN procedure in 740 inguinal basins [12] and Berger et al. evaluated this in 277 patients with H&N melanoma [23]. The next step will be to gather more data from the clinical performance of ICG-^99m^Tc-nanoscan to compare this to the previously studied ICG-^99m^Tc-nanocolloid populations.

## Conclusion

The outcomes of pre- and intraoperative SN visualization, ex vivo fluorescence measurements, lead to the conclusion that that ICG-^99m^Tc-nanoscan can safely replace ICG-^99m^Tc-nanocolloid as tracer for the SN procedure. However, due to the small study cohort, further performance studies are warranted.

## Supplementary information

Supplementary table 1 Distribution of SN in PeCa patients (Daseler’s classification) and H&N melanoma (level system of cervical LN classification). Level I: submandibular and submental region; level II: upper jugular nodes; level III: middle jugular nodes; level V: posterior triangle of the neck. H&N: head-and-neck; LN: lymph node. (DOCX 12 kb)

## Data Availability

The dataset used and/or analysed during the current study are available from the corresponding author on reasonable request.
