# Supplementary material for: Comparison of two hybrid sentinel node tracers: indocyanine green (ICG)-99mTc-nanocolloid vs. ICG-99mTc-nanoscan from a nuclear medicine and surgical perspective
Source: Eur J Nucl Med Mol Imaging. 2023 Mar 17;50(8):2282–91. doi: 10.1007/s00259-023-06157-9 (PMC10250462; doi:10.1007/s00259-023-06157-9)
Supplement: Supplementary file 1 — Distribution of SN in PeCa patients (Daseler’s classification) and H&N melanoma (level system of cervical LN classification). Level I: submandibular and submental region; level II: upper jugular nodes; level III: middle jugular nodes; level V: posterior triangle of the neck. H&N: head-and-neck; LN: lymph node. (DOCX 12 kb) [file 259_2023_6157_MOESM1_ESM.docx]

| Supplementary table 1 Distribution of SN in PeCa patients (Daseler’s classification) and H&N melanoma (level system of cervical LN classification) | |
| --- | --- |
|  | **Sentinel nodes on preoperative imaging (%)** |
| PeCa |  |
| Lateral superior | 15 (33) |
| Medial superior | 24 (52) |
| Central | 6 (13) |
| Lateral inferior | 1 (2) |
| Medial inferior | 0 |
|  |  |
| H&N melanoma |  |
| Level I | 2 (11) |
| Level II | 6 (32) |
| Level III | 3 (16) |
| Level IV, V, VI | 0 |
| Pre auricular | 2 (11) |
| Post auricular | 0 |
| Parotid | 6 (32) |
|  |  |
| Level I: submandibular and submental region; level II: upper jugular nodes; level III: middle jugular nodes; level V: posterior triangle of the neck. H&N: head and neck; LN: lymph node. | |
